# miR-139-5p Regulates the Proliferation of Acute Promyelocytic Leukemia Cells by Targeting MNT

**DOI:** 10.1155/2021/5522051

**Published:** 2021-04-16

**Authors:** Yueyue Fu, Limin Li, Jinxiao Hou, Huibo Li, Chengfang Lv, Hongjuan Yu, Xiaoqian Zhang, Mengyuan Xu, Mingwen Zhang, Hongbin Meng, Jie Liu, Xin Lian, Jiawei Feng, Jin Zhou

**Affiliations:** ^1^Department of Hematology, The First Affiliated Hospital, Harbin Medical University, Harbin 150001, Heilongjiang, China; ^2^Department of Hematology, South University of Science and Technology Hospital, Shenzhen, China

## Abstract

Acute promyelocytic leukemia (APL) patients with progressive leukocytosis are more likely to have various complications and poor outcomes. However, the regulatory roles of microRNAs in the leukocytosis of APL have not been clarified. Our study aims to evaluate the effects of miRNAs on leukocytosis during induction therapy of APL patients and explore its potential mechanisms. During induction treatment, patients with white blood cell count higher than 10 × 10^9^/L were divided into leukocytosis group and others were nonleukocytosis group. Using microarray assays, we found that miR-139-5p was significantly downregulated in the leukocytosis group. Elevated expression of miR-139-5p inhibited the proliferation of NB4 cells by arresting the cell cycle and inducing apoptosis. We further identified that MNT was a target of miR-139-5p. miR-139-5p significantly inhibited the proliferation, invasion, and migration function of NB4 cells through targeting MNT. Strategies for regulating miR-139-5p or MNT expression might provide new therapeutic approaches for progressive leukocytosis in APL.

## 1. Introduction

Acute promyelocytic leukemia (APL) is a distinct subtype of acute myeloid leukemia characterized by maturation arrest at the promyelocytic stage and life-threatening hemorrhages. Differentiation therapy using all-trans retinoic acid (ATRA) and/or arsenic trioxide (ATO) has significantly improved the prognosis of patients with APL [1–3]. However, early death and serious complications during induction treatment are still the most critical issues involved in the current care [4–6]. Our previous study shows that mortality of APL patients associated with ATO induction therapy was 16.7% mainly caused by hemorrhage, differentiation syndrome (DS), or infection [7].

White blood cell count at diagnosis is generally used for the risk stratification of patients with APL. High-risk patients (defined by WBC count > 10 × 10^9^/L on presentation) are more likely to have various complications and poor outcomes [8, 9]. Nevertheless, recent research demonstrates that progressive leukocytosis is also a relevant predictive marker for DS, early death, and subsequent relapse in APL [10]. Moreover, approximately 70% of non-high-risk patients treated with ATO develop leukocytosis during induction therapy despite treating with chemotherapy or leukapheresis [11]. Although differentiating APL cells should play a crucial role in the progression of white blood cell count, the precise genes and pathways that exert critical control remain unclear [12].

MicroRNAs (miRNAs) are endogenous small noncoding RNAs that play key regulatory roles in the biological processes of proliferation, differentiation, and apoptosis [13]. Dysregulation of miRNAs is involved in the pathogenesis of various cancers including hematologic malignancies [14, 15]. Several studies have identified a special expression profile of miRNAs in APL patients located in the human 14q32 imprinted domain and suggest that these miRNAs might play an important role in the molecular pathogenesis of APL [16–18]. A recent study showed that miR-382-5p inhibited differentiation of NB4 cell line by binding to PTEN which acts as a tumor suppressor and restores PML-containing nuclear bodies [19]. Liang et al. constructed a dysregulated microRNA network involved in ATO-induced apoptosis in APL. Their results indicated that ATO reduced the expression of let-7d and miR-766 through the degradation of PML and the inhibition of PML body recycling [20]. However, the regulatory functions of miRNAs in the progressive leukocytosis in APL patients have not been clarified. Our study aims to evaluate the effects of miRNAs on leukocytosis during induction therapy of APL and explore its potential molecular mechanisms of action.

## 2. Materials and Methods

### 2.1. Patient Sample

12 APL patients diagnosed in the First Affiliated Hospital of Harbin Medical University from January 2015 to June 2017 were enrolled in this study. The diagnosis of APL was confirmed by the demonstration of the t(15;17) chromosomal abnormality or promyelocytic leukemia-retinoic acid receptor (PML-RARA) rearrangements. All of these patients had WBC count lower than 10 × 10^9^/L at diagnosis. All patients received single-agent ATO (0.16 mg/kg/d, maximum 10 mg/day) for remission induction. When WBC count was 10 × 10^9^/L or higher, the ATO dose was reduced or discontinued temporarily and hydroxyurea (1.0–3.0 g/day) was administered to patients orally. Peripheral blood samples were obtained every day when ATO treatment started and mononuclear cells were isolated.

During induction treatment, patients with WBC counts higher than 10 × 10^9^/L were divided into leukocytosis group and others were nonleukocytosis group. Patient information is described in Table 1. The clinical features were comparable between the two groups, except the peak WBC count during therapy. For two groups, blood samples with the highest WBC count were selected. 12 blood samples, including 6 for the leukocytosis group and 6 for the nonleukocytosis group, were selected for the study. Detailed clinical and laboratory information is summarized in Supplemental Table 1.

### 2.2. Quantitative PCR-Based Array Experiments

TaqMan Low-Density Array (TLDA) on a 7900HT Real-Time PCR system was used for microRNA analysis. The quantitation expression of miRNA was detected using the TaqMan® Array Human MicroRNA A + B Cards Set v3.0 (Life Technologies, USA) with a total of 384 miRNAs and controls per card. Total RNA was isolated using mirVana miRNA Isolation Kit and reverse-transcribed using the TaqMan® MicroRNA Reverse Transcription Kit (both Life Technologies, USA). miRNA expression profiling was performed according to the manufacturer's instructions.

SDS 2.4 and the ExpressionSuite v1.0.1. software (Life Technologies, USA) were used for processing and evaluation. From the detected Ct values, the relative quantity was calculated using the 2^-Ct^ formula. *T*-test in combination with the Pavlidis Template Matching tool (TIGR TM4 suite) was used to filter the differently expressed miRNAs. The *P* value (*P* < 0.05) and fold-change (FC > 3) thresholds were set in the comparison of miRNAs in different groups of samples.

### 2.3. Quantitative Real-Time PCR Assay

Total RNA was extracted using the TRIzol reagent (Invitrogen) following the manufacturer's instructions and reverse-transcribed into cDNA using Superscript III Reverse Transcriptase (Invitrogen, USA). Quantitative real-time PCR (qRT-PCR) was performed using a TaqMan miRNA kit (Life Technologies, USA) using the ABI PRISM 7900 HT sequence detection system (Applied Biosystems, CA). U6 was used as the internal control. The following primers were used for real-time amplification: miR-139-5p (forward 5′-TCTACAGTGCACGTGTC-3′, reverse 5′-GAATACCTCGGACCCTGC-3′) and U6 (forward 5′-CTCGCTTCGGCAGCACA-3′, reverse 5′-AACGCTTCACGAATTTGCGT-3'). The relative expression of miR-139-5p was measured by the 2^−ΔΔCt^ method.

### 2.4. Luciferase Reporter Assay

Target genes of miR-139-5p were obtained from MiRanda (http://www.MicroRNA.org/Miranda), TargetScan (www. genes.mit.edu/targetscan), miRDB (http://mirdb.org/miRDB/), and PicTar (http://www.pictar.bio.nyu.edu). Among the targets predicted, MAX-binding protein (MNT) was selected for our study. The MNT-3′UTR-wild-type or mutant sequence (MNT-3′UTRM) was cloned to the pGL3 luciferase reporter vector (Promega, USA). Wild-type or mutant vector was cotransfected with miR-139-5p or control into 293T cells using Lipofectamine 2000 (Invitrogen, USA). After 48 h, luciferase activities were measured using the Dual-Luciferase Reporter Assay System kit (Promega, USA). Renilla luciferase activity was used as the normalization.

### 2.5. Cell Culture and Transfection

The human acute promyelocytic leukemia cell lines NB4 were a gift from Tianjin Institute of Hematology, Chinese Academy of Medical Sciences. The cells were maintained in RPMI 1640 culture medium (Gibco, USA) containing 10% fetal bovine serum (Gibco, USA) and incubated at 37°C and 5% CO_2_ in a humid environment.

The miR-139-5p mimics, mimics negative controls, and miR-139-5p mimics + pEGFP-MNT were purchased from GenePharma (Shanghai, China). The sequences were as follows: miR-139-5p mimics, UCUACAGUGCACGUGUCUCCAGU and UGGAGACACGUGCACUGUAGAUU; negative control (NC), UUCUCCGAACGUGUCACGUTT and ACGUGACACGUUCGGAGAATT; transfections were performed using Lipofectamine 2000 (Invitrogen) according to the manufacturer's recommended protocol. After 48 h of transfection, the cells were harvested for further experiments. The transfection efficiency of miR-139-5p or MNT was confirmed by detecting the microRNA or protein expression levels using qRT-PCR or western blotting.

### 2.6. Western Blotting

Mononuclear cells or NB4 cells were lysed in RIPA protein lysis buffer with protease inhibitor (Beyotime, China). The protein concentration was quantified using a BCA protein assay kit (Beyotime, China). The same amounts of proteins from each sample were loaded on SDS-PAGE and transferred to the PVDF membrane. MNT (Abcam, China) and *β*-actin (Beijing Zhongshan Golden Bridge Biotechnology, China) were used as primary antibodies. The resulting bands were visualized using an ECL kit (Millipore, USA). The results were quantified using Gel-Pro-Analyzer.

### 2.7. Cell Proliferation Assay

Cell Counting Kit-8 (CCK-8) assay was used to detect cell proliferation, according to the manufacturer's instructions. NB4 cells were plated into 96-well plates and transfected. After incubation for 0, 24, 48, or 72 h, CCK-8 reagent (Solarbio, China) was added into each well and incubated for 2 hours at 37°C. Absorbance was measured at 450 nm using a microplate reader (ELX-800, BioTek Instruments, USA).

### 2.8. Cell Cycle and Apoptosis Assay

For cell cycle analysis, the transfected NB4 cells were gathered by trypsinization and fixed in 70% ethanol at 4°C overnight. Cells were resuspended and stained with 50 *μ*g/mL propidium iodide (BD Biosciences, USA) containing RNase A for 20–30 minutes at room temperature in the dark. Apoptosis was determined by dual staining with Annexin V-Light 650 and propidium iodide (BD Biosciences, USA). In brief, cells were collected after transfected and stained with Annexin V-Light 650 and propidium iodide according to the manufacturer's instructions. Cells were analyzed using FACSCalibur flow cytometer (BD Biosciences, USA).

### 2.9. Transwell Invasion and Migration Assays

Cell invasion was performed by Matrigel invasion assay (BD Biosciences, USA). Transfected NB4 cells were harvested, suspended (1 × 10^5^/well) in 500 uL serum-free medium, and then added to the upper chamber, while 600 *μ*L DMEM containing 20% FBS was added to the lower chamber. After 24 h incubation at 37°C, cells that had invaded through the Matrigel membrane were stained with trypan blue and counted (four high-power fields, ×100 magnification). Transwell migration assay was carried out by the same method, but the transwell chambers used were not coated with Matrigel.

### 2.10. Statistical Analysis

All results were obtained from at least three independent experiments. The data were shown as mean ± SD. Statistical comparisons were evaluated using Student's *t*-test or ANOVA with SPSS 22.0. *P* value less than 0.05 was taken as statistically significant.

## 3. Results

### 3.1. miR-139-5p Was Significantly Downregulated in the Leukocytosis Group

**Figure 1:**
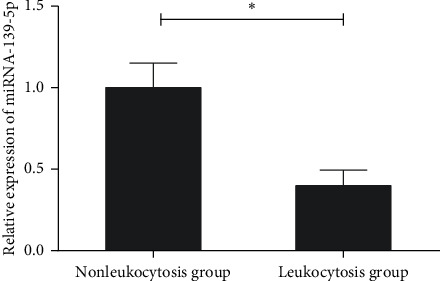
The expressions of miR-139-5p in the leukocytosis and nonleukocytosis groups. qRT-PCR was performed to analyse the miR-139-5p expression levels. Three independent experiments were performed. ^*∗*^*P* < 0.05.

miRNA expression profilings were analyzed in 12 samples, including 6 for the leukocytosis group and 6 for the nonleukocytosis group. Data of the quantitative PCR-based array is shown in Supplemental Table 2. miRNAs with fold change (FC > 3) and *P* value (*P* < 0.05) were considered significantly differentially expressed. A total of 9 miRNAs were selected, including 4 upregulated miRNAs and 5 downregulated miRNAs in the leukocytosis group (Table 2). Among the 9 microRNAs, miR-139-5p had been reported to be a tumor suppressor in multiple cancers and inhibit protein translation in acute myeloid leukemia. To validate the miRNA quantitative PCR-based array data, the expression of miR-139-5p was analyzed by RT-qPCR using the samples analyzed on the array. Consistent with the results of the quantitative PCR-based array, miR-139-5p was significantly downregulated in the leukocytosis group (Figure 1). This finding raised the possibility that miR-139-5p might play an important role in the development of leukocytosis of APL patients.

### 3.2. MNT Was a Target of miR-139-5p

**Figure 2:**
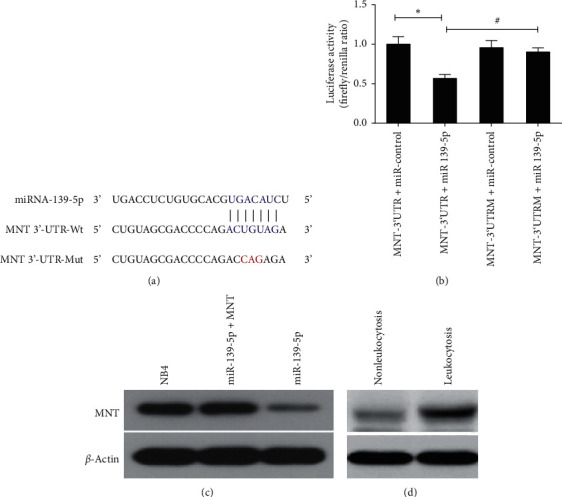
The interaction between miR-139-5p and MNT. (a) Predicted binding sites for miR-139-5p and MNT. The sequence of the mutant 3′-UTR of MNT is presented. (b) Luciferase assays showed a decrease in relative luciferase activity in 293T cells cotransfected with miR-139-5p and MNT. Mutated 3′-UTR MNT plasmid was used as the control. (c) The protein levels of MNT in NB4 cells transfected with or without miR-139-5p or combined with MNT were evaluated by western blot analysis. (d) The protein levels of MNT in APL patients of leukocytosis and nonleukocytosis groups were evaluated by western blot analysis. Three independent experiments were performed. ^*∗*,#^*P* < 0.05.

TargetScan, MiRanda, PicTar, and miRDB were used to search potential targets of miR139-5p. Among the predicted targets, MAX-binding protein (MNT) was selected as a putative target for its relevance to myeloid differentiation [21]. To demonstrate the interaction between miR-139-5p and MNT, luciferase reporters containing the wild-type or mutant 3′ UTR of MNT were constructed (Figure 2(a)). Cotransfection of miR-139-5p and the luciferase reporter with wild-type MNT 3′-UTR greatly reduced the luciferase activity. However, no significant difference was found when cotransfected with the mutant 3′-UTR (Figure 2(b)). Next, to explore the regulatory effect of miR-139-5p on MNT expression, NB4 cells were transfected with or without miR-139-5p mimics or negative controls or with MNT. Then the expression level of MNT protein was detected by western blot. Significant reduction of MNT protein levels in NB4 cells was detected after transfecting with miR-139-5p mimics and was remarkably restored by MNT overexpression (Figure 2(c)). These results indicate that miR-139-5p directly binds to the 3′ UTR of MNT and significantly downregulates its protein expression. We further detected the MNT protein levels of APL patients in leukocytosis and nonleukocytosis groups. The results showed that there was a dramatic increase in the expression level of MNT protein in leukocytosis APL patients (Figure 2(d)).

### 3.3. miR-139-5p Inhibited Cell Proliferation through Targeting MNT

**Figure 3:**
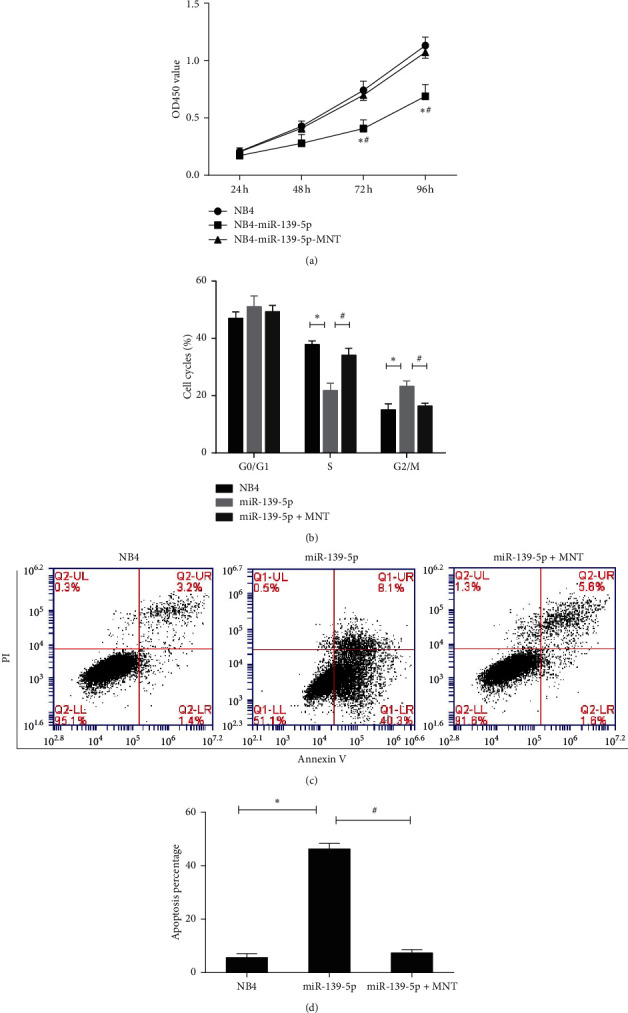
The effect of miR-139-5p on cell proliferation of NB4 cells. (a) Cell proliferation of the transfected NB4 cells was detected by CCK-8 assay at 24, 48, 72, and 96 h. (b) Cell cycle distribution analysis was detected using flow cytometry in the transfected NB4 cells. (c, d) Apoptosis of NB4 cells was detected using flow cytometry. Three independent experiments were performed. ^*∗*^*P* < 0.05: miR-139-5p versus NB4; ^#^*P* < 0.05: miR-139-5p versus miR-139-5p-MNT.

To evaluate the effect of miR-139-5p on cell proliferation, NB4 cells were transfected with or without miR-139-5p or along with MNT. The CCK-8 assay manifested that miR-139-5p mimics remarkably inhibited the proliferation of NB4 cells, while this effect was reversed after forced expression of MNT (Figure 3(a)). Cell cycle and apoptosis were examined to investigate the mechanisms through which miR-139-5p inhibits cell growth. As shown in Figure 3(b), NB4 cells transfected with miR-139-5p showed an obvious decrease of the cells in the S phase and an increase of that in the G2/M phase. The arrest of the cell cycle caused by transfected miR-139-5p was alleviated by the expression of MNT. Besides that, the number of apoptotic cells increased in response to the miR-139-5p but decreased with the transfection of miR-139-5p-MNT (Figures 3(c) and 3(d)). These data demonstrated that miR-139-5p inhibits the growth of NB4 cells by arresting the cell cycle in the G2/M phase and promoting apoptosis through targeting MNT.

### 3.4. miR-139-5p Suppressed Cell Invasion and Migration through Targeting MNT

**Figure 4:**
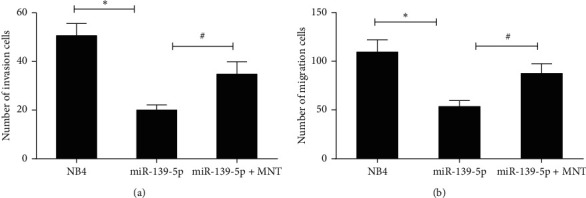
The effects of miR-139-5p on cell invasion and migration in NB4 cells. NB4 cells were transfected with or without miR-139-5p or along with MNT. Transwell invasion (a) and migration (b) assays were carried out to detect the invasion and migration ability of the transfected NB4 cells. Three independent experiments were performed. ^*∗*^*P* < 0.05: miR-139-5p versus NB4; ^#^*P* < 0.05: miR-139-5p versus miR-139-5p-MNT.

We further assessed the effects of miR-139-5p on the invasion and migration of NB4 cells. Transwell invasion and migration assays showed that increased expression of miR-139-5p significantly repressed the invasion and migration ability of cells (Figures 4(a) and 4(b)). In contrast, forced expression of MNT rectified the effects of miR-139-5p on reducing the numbers of invasion and migration cells. These results evaluated that miR-139-5p inhibited cell invasion and migration through targeting MNT.

## 4. Discussion

ATO, an ancient traditional Chinese medicine, has been successfully applied to treat APL. It is in our hospital that ATO was initially attempted for clinical treatment of APL in the 1970s [22]. Nowadays, single-agent ATO has been proven to be highly effective in the treatment of APL [23–25]. The molecular mechanism of the antileukemic effect of ATO is complex. Studies have demonstrated that ATO exerts dual effects on APL cells mainly through the induction of apoptosis and partial differentiation [26]. Despite the predominant effect of ATO, leukocytosis which is related to severe complications and poor outcomes during induction therapy is still an unsolved problem. Although differentiating in APL cells or some cytokines and adhesion molecules were so far indicated to participate in the leukocytosis of APL patients, no definite explanation for such a proliferative effect has been demonstrated clearly [12]. In this study, the effects of miRNAs on leukocytosis of APL patients were explored.

APL patients with WBC count < 10 × 10^9^/L at diagnosis were enrolled in this study. During the induction therapy of ATO, patients with maximum leukocyte count higher than 10 × 10^9^/L were stratified into the leukocytosis group. Using quantitative PCR-based array assay, a total of 9 miRNAs were found to be differentially expressed. Among them, miR-139-5p was significantly downregulated in the leukocytosis group. miRNA-139-5p, located in 11q13.4, has been well demonstrated to regulate diverse biological processes, such as proliferation, invasion, and cell death [27]. Previous studies showed that as a tumor suppressor, miRNA-139-5p was downregulated in multiple carcinomas, such as bladder cancer, breast cancer, and colorectal cancer [28–30]. Additionally, its decreased expression is also correlated with poor prognosis and may serve as a promising biomarker in cancer development and progression [27]. Moreover, miR-139-5p was also found to be related to hematopoietic malignancy. A recent study revealed that elevated expression of miR-139-5p inhibits protein translation in acute myeloid leukemia and was associated with a favorable outcome of patients [31]. Choi et al. exhibited that overexpression of miR-139-5p inhibited the proliferation of hematopoietic progenitors and resulted in the remission of chronic myeloid leukemia [32]. However, the physiological function and molecular mechanism of miR-139-5p in the leukocytosis of APL patients still remain unknown.

In the current study, we established that miR-139-5p inhibited the proliferation of NB4 cells by arresting the cell cycle and inducing apoptosis. Besides that, functional analyses showed that increased expression of miR-139-5p also inhibited the invasion and migration function of NB4 cells. These results indicate the critical role of miR-139-5p in the formation of leukocytosis in APL patients. We further identified that MNT was a target of miR-139-5p. Elevated expression of miR-139-5p significantly downregulated the expression of MNT protein and suppressed the proliferation, invasion, and migration of NB4 cells through targeting MNT.

MNT is a member of the Myc/Max/Mad transcription factors network which regulates cell proliferation, differentiation, and transformation. MNT heterodimerizes with MAX and acts as a tumor suppressor [33]. Nilsson et al. demonstrated that as an MYC antagonist, MNT loss promoted proliferation, transformation, and sensitivity to apoptotic stimuli, which are characteristics of MYC overexpression [34]. However, other studies found that MNT functioned as a facilitator of proliferation and oncogenesis driven by MYC rather than a tumor suppressor [35]. Increasing expression of MNT in highly proliferating cells confers prosurvival activity through PI3 kinase signaling by repressing genes that promote apoptosis. Consistent with this finding, deletion of MNT in the MYC-driven T cell lymphoma model resulted in increased apoptosis and reduced tumorigenesis [36]. In our study, we found that MNT acted as a promoter of cell proliferation. Suppression of MNT protein expression inhibited the invasion and migration function of NB4 cells. Nevertheless, the precise regulatory networks of MNT in the process of leukocytosis in APL need to be further explored.

This study provides insights into the mechanisms involved in the leukocytosis of APL patients receiving ATO induction therapy. Our findings confirmed that miR-139-5p was downregulated in the leukocytosis group of APL patients. Elevated expression of miR-139-5 significantly inhibited the proliferation, invasion, and migration function of NB4 cells through targeting MNT. These results indicate the critical role of miR-139-5p and MNT in the formation of leukocytosis in APL patients. Strategies for regulating miR-139-5p or MNT expression might provide the potential for new therapeutic methods.

## Figures and Tables

**Table 1:** Clinical characteristics of APL patients.

Patient	Gender	Age	WBC count at diagnosis (×10^9^/L)	Peak WBC count (×10^9^/L)
Nonleukocytosis group				
1	F	36	3.6	7.0
2	M	50	0.4	2.8
3	F	29	1.3	5.1
4	F	41	0.6	3.4
5	M	56	1.0	7.9
6	M	27	3.7	8.3

Leukocytosis group				
1	F	44	4.4	65.4
2	F	64	5.1	20.1
3	F	36	0.9	33.8
4	M	25	2.6	43.7
5	M	51	1.3	53.8
6	F	19	0.7	23.6

M: male; F: female; WBC: white blood cell.

**Table 2:** Differentially expressed miRNAs between the leukocytosis group and the nonleukocytosis group.

Upregulated miRNAs	*P*	Fold change	Downregulated miRNAs	*P*	Fold change
hsa-miR-193a-5p	0.014	3.54	hsa-miR-139-5p	<0.001	−6.39
hsa-miR-361-5p	0.022	11.49	hsa-miR-616-3p	0.047	−4.87
hsa-miR-379-5p	0.023	10.45	hsa-miR-549	0.019	−8.86
hsa-miR-542-5p	0.043	9.35	hsa-miR-144-3p	0.046	−12.3
			hsa-miR-1305	0.024	−5.44

## Data Availability

All data generated or analyzed during this study are available from the corresponding author on reasonable request.

## Supplementary Materials

Supplementary Materials Supplemental Table 1: clinical and laboratory information of patients. Supplemental Table 2: data of the quantitative PCR-based array.
