# Supplementary material for: miR-139-5p Regulates the Proliferation of Acute Promyelocytic Leukemia Cells by Targeting MNT
Source: J Oncol. 2021 Apr 16;2021:5522051. doi: 10.1155/2021/5522051 (PMC8064781; doi:10.1155/2021/5522051)
Supplement: Supplementary Materials — Supplemental Table 1: clinical and laboratory information of patients. Supplemental Table 2: data of the quantitative PCR-based array. [file 5522051.f1.zip › 5522051.f1/Supplemental Table 2.docx]

**Supplemental Table 2. Data of the quantitative PCR-based array.**

| Assay | Fold Difference | p-value | Fold change |
| --- | --- | --- | --- |
| hsa-let-7a | 0.35 | 0.6245 | -2.88 |
| hsa-let-7c | 0.95 | 0.9494 | -1.05 |
| hsa-let-7d | 0.77 | 0.6739 | -1.30 |
| hsa-let-7e | 1.17 | 0.8240 | 1.17 |
| hsa-let-7f | 0.58 | 0.7529 | **-1.74** |
| hsa-let-7g | 0.76 | 0.6769 | -1.31 |
| hsa-miR-1 | 1.93 | 0.2472 | 1.93 |
| hsa-miR-9 | 1.46 | 0.5270 | 1.46 |
| hsa-miR-10a | 0.43 | 0.4383 | -2.34 |
| hsa-miR-10b | 1.93 | 0.2472 | 1.93 |
| U6 snRNA-1 | 1.00 | 0.9852 | -1.00 |
| U6 snRNA-2 | 1.03 | 0.7319 | 1.03 |
| hsa-miR-15a | **0.04** | 0.0717 | **-24.92** |
| hsa-miR-15b | 0.41 | 0.7665 | -2.44 |
| hsa-miR-16 | 0.74 | 0.4428 | -1.36 |
| hsa-miR-17 | 0.38 | 0.2530 | -2.64 |
| hsa-miR-18a | **0.27** | 0.1647 | **-3.67** |
| hsa-miR-18b | 1.99 | 0.4012 | 1.99 |
| hsa-miR-19a | 0.61 | 0.3089 | -1.63 |
| hsa-miR-19b | 0.39 | 0.2740 | -2.54 |
| hsa-miR-20a | **0.30** | 0.3059 | **-3.34** |
| hsa-miR-20b | **0.22** | 0.2718 | **-4.48** |
| hsa-miR-21 | 0.36 | 0.1989 | -2.79 |
| hsa-miR-22 | **0.33** | 0.5708 | **-3.06** |
| hsa-miR-23a | 0.99 | 0.9886 | -1.01 |
| hsa-miR-23b | 2.46 | 0.2299 | 2.46 |
| hsa-miR-24 | 0.49 | 0.4560 | -2.05 |
| hsa-miR-25 | **0.23** | 0.2133 | **-4.29** |
| hsa-miR-26a | 0.76 | 0.5184 | -1.31 |
| hsa-miR-26b | 1.12 | 0.6820 | 1.12 |
| hsa-miR-27a | **0.25** | 0.1709 | **-3.93** |
| hsa-miR-27b | 1.57 | 0.6841 | 1.57 |
| hsa-miR-28-3p | **0.30** | 0.6147 | **-3.28** |
| hsa-miR-28 | **0.25** | 0.2411 | **-4.07** |
| U6 snRNA-3 | 1.03 | 0.6631 | 1.03 |
| U6 snRNA-4 | 0.95 | 0.4624 | -1.06 |
| hsa-miR-29a | 0.36 | 0.1960 | -2.74 |
| hsa-miR-29b | 0.34 | 0.1403 | -2.90 |
| hsa-miR-29c | **0.11** | 0.2837 | **-9.16** |
| hsa-miR-30b | 0.50 | 0.5151 | -2.01 |
| hsa-miR-30c | 0.47 | 0.4988 | -2.13 |
| hsa-miR-31 | **8.60** | 0.1627 | **8.60** |
| hsa-miR-32 | 1.11 | 0.9248 | 1.11 |
| hsa-miR-33b | 1.93 | 0.2472 | 1.93 |
| hsa-miR-34a | **0.15** | 0.2395 | **-6.76** |
| hsa-miR-34c | 1.93 | 0.2472 | 1.93 |
| hsa-miR-92a | 0.94 | 0.9188 | -1.06 |
| mmu-miR-93 | **0.23** | 0.2472 | **-4.31** |
| hsa-miR-95 | **3.63** | 0.1252 | **3.63** |
| mmu-miR-96 | 1.93 | 0.2472 | 1.93 |
| hsa-miR-98 | 0.72 | 0.8152 | -1.40 |
| hsa-miR-99a | 0.52 | 0.4123 | -1.92 |
| hsa-miR-99b | 1.82 | 0.4708 | 1.82 |
| hsa-miR-100 | **0.32** | 0.2217 | **-3.17** |
| hsa-miR-101 | **0.18** | 0.1011 | **-5.48** |
| hsa-miR-103 | 0.62 | 0.4784 | -1.62 |
| hsa-miR-105 | 1.93 | 0.2472 | 1.93 |
| hsa-miR-106a | 0.37 | 0.2467 | -2.71 |
| RNU44 | 0.84 | 0.8157 | -1.19 |
| hsa-miR-106b | 0.41 | 0.2178 | -2.47 |
| hsa-miR-107 | 1.38 | 0.6546 | 1.38 |
| hsa-miR-122 | **3.11** | 0.2365 | **3.11** |
| mmu-miR-124a | 0.39 | 0.2318 | -2.54 |
| hsa-miR-125a-3p | 2.61 | **0.0194** | 2.61 |
| hsa-miR-125a-5p | 1.58 | 0.7439 | 1.58 |
| hsa-miR-125b | **0.29** | 0.2329 | **-3.39** |
| hsa-miR-126 | 0.83 | 0.7557 | -1.21 |
| hsa-miR-127 | **0.12** | 0.1607 | **-8.58** |
| hsa-miR-127-5p | 1.93 | 0.2472 | 1.93 |
| hsa-miR-128a | 0.51 | 0.2870 | -1.96 |
| mmu-miR-129-3p | 1.93 | 0.2472 | 1.93 |
| hsa-miR-129 | 1.93 | 0.2472 | 1.93 |
| hsa-miR-130a | 0.86 | 0.9050 | -1.16 |
| hsa-miR-130b | **3.11** | 0.4946 | **3.11** |
| hsa-miR-132 | **0.31** | 0.6199 | **-3.28** |
| hsa-miR-133a | 1.13 | 0.9080 | 1.13 |
| hsa-miR-133b | 1.93 | 0.2472 | 1.93 |
| mmu-miR-134 | 0.49 | 0.5090 | -2.05 |
| hsa-miR-135a | 1.93 | 0.2472 | 1.93 |
| hsa-miR-135b | 1.93 | 0.2472 | 1.93 |
| hsa-miR-136 | 0.39 | 0.5671 | -2.59 |
| mmu-miR-137 | 1.04 | 0.9655 | 1.04 |
| hsa-miR-138 | 2.08 | 0.2364 | 2.08 |
| hsa-miR-139-3p | 1.93 | 0.2472 | 1.93 |
| hsa-miR-139-5p | **6.39** | **0.0009** | **6.39** |
| hsa-miR-140-3p | 1.49 | 0.3570 | 1.49 |
| mmu-miR-140 | **0.29** | 0.2281 | **-3.50** |
| hsa-miR-141 | 0.85 | 0.8025 | -1.18 |
| hsa-miR-142-3p | **0.14** | 0.2837 | **-7.36** |
| hsa-miR-142-5p | 0.35 | 0.1568 | -2.86 |
| hsa-miR-143 | 0.40 | 0.3399 | -2.48 |
| hsa-miR-145 | 0.80 | 0.7964 | -1.26 |
| hsa-miR-146a | 0.68 | 0.5326 | -1.47 |
| hsa-miR-146b-3p | 1.95 | 0.2314 | 1.95 |
| hsa-miR-146b | 2.04 | 0.3884 | 2.04 |
| hsa-miR-147b | 1.93 | 0.2472 | 1.93 |
| hsa-miR-148a | 0.89 | 0.7863 | -1.12 |
| hsa-miR-148b | 1.69 | 0.5762 | 1.69 |
| hsa-miR-149 | 0.86 | 0.8782 | -1.16 |
| hsa-miR-150 | **4.40** | 0.2448 | **4.40** |
| hsa-miR-152 | 0.50 | 0.3565 | -1.99 |
| mmu-miR-153 | 1.93 | 0.2472 | 1.93 |
| hsa-miR-154 | 1.93 | 0.2472 | 1.93 |
| hsa-miR-181a | 0.70 | 0.7484 | -1.43 |
| hsa-miR-181c | 1.50 | 0.8172 | 1.50 |
| hsa-miR-182 | 2.48 | 0.0766 | 2.48 |
| RNU48 | 1.53 | 0.4681 | 1.53 |
| hsa-miR-183 | 1.93 | 0.2472 | 1.93 |
| hsa-miR-184 | 0.38 | 0.5031 | -2.64 |
| hsa-miR-185 | 0.35 | 0.1743 | -2.85 |
| hsa-miR-186 | 1.00 | 0.9975 | -1.00 |
| mmu-miR-187 | 1.88 | 0.8983 | 1.88 |
| hsa-miR-188-3p | **0.16** | 0.5063 | **-6.19** |
| hsa-miR-190 | 1.42 | 0.5669 | 1.42 |
| hsa-miR-191 | 1.22 | 0.8551 | 1.22 |
| hsa-miR-192 | 0.79 | 0.8782 | -1.26 |
| hsa-miR-193a-3p | **0.11** | 0.3732 | **-8.79** |
| hsa-miR-193a-5p | **0.28** | **0.0141** | **-3.54** |
| hsa-miR-193b | 0.64 | 0.7215 | -1.56 |
| hsa-miR-194 | **0.28** | 0.3521 | **-3.51** |
| hsa-miR-195 | 0.89 | 0.9254 | -1.12 |
| hsa-miR-196b | 2.16 | 0.0657 | 2.16 |
| hsa-miR-197 | 1.65 | 0.5523 | 1.65 |
| hsa-miR-198 | 1.93 | 0.2472 | 1.93 |
| hsa-miR-199a | 1.74 | 0.3220 | 1.74 |
| hsa-miR-199a-3p | 0.57 | 0.6830 | -1.74 |
| hsa-miR-199b | **0.32** | 0.2593 | **-3.12** |
| hsa-miR-200a | 0.76 | 0.8015 | -1.32 |
| hsa-miR-200b | **3.84** | 0.3197 | **3.84** |
| hsa-miR-200c | 1.50 | 0.5464 | 1.50 |
| hsa-miR-202 | **0.10** | 0.3330 | **-9.74** |
| hsa-miR-203 | 0.97 | 0.9637 | -1.03 |
| hsa-miR-204 | 1.93 | 0.2472 | 1.93 |
| hsa-miR-205 | 0.90 | 0.9083 | -1.11 |
| hsa-miR-208b | 1.93 | 0.2472 | 1.93 |
| hsa-miR-210 | 1.36 | 0.7334 | 1.36 |
| hsa-miR-214 | 0.67 | 0.6626 | -1.49 |
| hsa-miR-215 | 1.93 | 0.2472 | 1.93 |
| hsa-miR-216a | 1.52 | 0.4198 | 1.52 |
| hsa-miR-216b | 0.47 | 0.3869 | -2.14 |
| hsa-miR-217 | 1.52 | 0.4189 | 1.52 |
| hsa-miR-218 | 1.40 | 0.4807 | 1.40 |
| hsa-miR-219 | 0.86 | 0.8755 | -1.17 |
| hsa-miR-221 | 0.49 | 0.4392 | -2.06 |
| hsa-miR-222 | 0.47 | 0.4603 | -2.12 |
| hsa-miR-223 | 0.89 | 0.7831 | -1.13 |
| hsa-miR-224 | 0.60 | 0.5668 | -1.68 |
| hsa-miR-296-3p | 1.93 | 0.2472 | 1.93 |
| hsa-miR-296 | 1.93 | 0.2471 | 1.93 |
| hsa-miR-299-3p | 1.93 | 0.2472 | 1.93 |
| hsa-miR-299-5p | 1.50 | 0.4933 | 1.50 |
| hsa-miR-301 | 0.52 | 0.4035 | -1.92 |
| hsa-miR-301b | 0.47 | 0.0730 | -2.12 |
| hsa-miR-302a | 0.56 | 0.6794 | -1.78 |
| ath-miR159a | 1.93 | 0.2472 | 1.93 |
| hsa-miR-302b | 1.93 | 0.2472 | 1.93 |
| hsa-miR-302c | 1.97 | 0.6242 | 1.97 |
| hsa-miR-320 | 0.95 | 0.9140 | -1.05 |
| hsa-miR-323-3p | 1.07 | 0.9462 | 1.07 |
| hsa-miR-324-3p | 0.45 | 0.3513 | -2.20 |
| hsa-miR-324-5p | **0.25** | 0.2355 | **-4.01** |
| hsa-miR-326 | 1.93 | 0.2472 | 1.93 |
| hsa-miR-328 | 1.04 | 0.9354 | 1.04 |
| hsa-miR-329 | 1.93 | 0.2472 | 1.93 |
| hsa-miR-330 | 0.86 | 0.8393 | -1.16 |
| hsa-miR-330-5p | 1.95 | 0.2450 | 1.95 |
| hsa-miR-331 | 0.52 | 0.4851 | -1.92 |
| hsa-miR-331-5p | 0.85 | 0.9307 | -1.18 |
| hsa-miR-335 | 0.59 | 0.5166 | -1.68 |
| hsa-miR-337-5p | 0.43 | 0.5336 | -2.31 |
| hsa-miR-338-3p | 0.37 | 0.4271 | -2.69 |
| hsa-miR-339-3p | 0.48 | 0.5414 | -2.09 |
| hsa-miR-339-5p | 0.55 | 0.6363 | -1.81 |
| hsa-miR-340 | 0.83 | 0.7521 | -1.20 |
| hsa-miR-155 | 2.01 | 0.4505 | 2.01 |
| hsa-let-7b | **0.14** | 0.2164 | **-7.14** |
| hsa-miR-342-3p | **5.04** | 0.2076 | **5.04** |
| hsa-miR-342-5p | 1.51 | 0.4846 | 1.51 |
| hsa-miR-345 | 1.37 | 0.4126 | 1.37 |
| hsa-miR-361 | **0.09** | **0.0227** | **-11.49** |
| hsa-miR-362-3p | 1.12 | 0.8816 | 1.12 |
| hsa-miR-362 | 0.54 | 0.3243 | -1.86 |
| hsa-miR-363 | 1.83 | 0.0663 | 1.83 |
| hsa-miR-365 | 1.55 | 0.7869 | 1.55 |
| hsa-miR-367 | 1.93 | 0.2472 | 1.93 |
| hsa-miR-369-3p | 1.47 | 0.5151 | 1.47 |
| hsa-miR-369-5p | 1.93 | 0.2472 | 1.93 |
| hsa-miR-370 | 0.54 | 0.5954 | -1.85 |
| hsa-miR-371-3p | 1.93 | 0.2472 | 1.93 |
| hsa-miR-372 | 1.93 | 0.2472 | 1.93 |
| hsa-miR-373 | 0.97 | 0.9649 | -1.03 |
| hsa-miR-374 | 0.39 | 0.3083 | -2.56 |
| mmu-miR-374-5p | **0.23** | 0.4333 | **-4.43** |
| hsa-miR-375 | 1.93 | 0.2472 | 1.93 |
| hsa-miR-376a | **0.16** | 0.4073 | **-6.44** |
| hsa-miR-376b | 1.93 | 0.2472 | 1.93 |
| hsa-miR-377 | 1.93 | 0.2472 | 1.93 |
| mmu-miR-379 | **0.10** | **0.0231** | **-10.45** |
| hsa-miR-380-3p | 1.93 | 0.2472 | 1.93 |
| hsa-miR-381 | 1.93 | 0.2472 | 1.93 |
| hsa-miR-382 | 0.49 | 0.6416 | -2.06 |
| hsa-miR-383 | 1.93 | 0.2472 | 1.93 |
| hsa-miR-409-5p | 1.93 | 0.2472 | 1.93 |
| hsa-miR-410 | 0.36 | 0.2536 | -2.75 |
| hsa-miR-411 | **0.10** | 0.1873 | **-10.30** |
| hsa-miR-422a | 0.90 | 0.9065 | -1.11 |
| hsa-miR-423-5p | 0.36 | 0.2936 | -2.77 |
| hsa-miR-424 | **0.26** | 0.2754 | **-3.84** |
| hsa-miR-425-5p | 1.33 | 0.4882 | 1.33 |
| hsa-miR-429 | 1.93 | 0.2472 | 1.93 |
| hsa-miR-431 | 0.83 | 0.9138 | -1.20 |
| hsa-miR-433 | 0.75 | 0.6603 | -1.33 |
| hsa-miR-449 | 0.95 | 0.9500 | -1.05 |
| hsa-miR-449b | 1.93 | 0.2472 | 1.93 |
| hsa-miR-450a | 1.16 | 0.9081 | 1.16 |
| hsa-miR-450b-3p | 1.93 | 0.2472 | 1.93 |
| hsa-miR-450b-5p | **6.21** | 0.2074 | **6.21** |
| mmu-miR-451 | 1.64 | 0.4273 | 1.64 |
| hsa-miR-452 | **0.29** | 0.2470 | **-3.46** |
| hsa-miR-453 | 1.93 | 0.2472 | 1.93 |
| hsa-miR-454 | 1.04 | 0.9622 | 1.04 |
| hsa-miR-455-3p | 1.93 | 0.2472 | 1.93 |
| hsa-miR-455 | 1.93 | 0.2472 | 1.93 |
| hsa-miR-483-5p | 1.74 | 0.7375 | 1.74 |
| hsa-miR-484 | 1.97 | 0.4177 | 1.97 |
| hsa-miR-485-3p | 1.85 | 0.3383 | 1.85 |
| hsa-miR-485-5p | 1.93 | 0.2472 | 1.93 |
| hsa-miR-486-3p | 1.63 | 0.7116 | 1.63 |
| hsa-miR-486 | 2.31 | 0.5851 | 2.31 |
| hsa-miR-487a | 0.48 | 0.6042 | -2.09 |
| hsa-miR-487b | **0.19** | 0.1136 | **-5.18** |
| hsa-miR-488 | 0.95 | 0.9457 | -1.05 |
| hsa-miR-489 | 1.20 | 0.7621 | 1.20 |
| hsa-miR-490 | 1.93 | 0.2472 | 1.93 |
| hsa-miR-491-3p | 1.93 | 0.2472 | 1.93 |
| mmu-miR-491 | **0.29** | 0.2509 | **-3.42** |
| hsa-miR-493 | 0.54 | 0.4535 | -1.87 |
| hsa-miR-494 | **0.18** | 0.0856 | **-5.56** |
| mmu-miR-495 | 0.34 | 0.1215 | -2.98 |
| mmu-miR-496 | 1.93 | 0.2472 | 1.93 |
| hsa-miR-499-3p | 1.93 | 0.2472 | 1.93 |
| mmu-miR-499 | 1.93 | 0.2472 | 1.93 |
| hsa-miR-500 | 0.51 | 0.5091 | -1.97 |
| hsa-miR-501-3p | 1.93 | 0.2472 | 1.93 |
| hsa-miR-501 | 0.34 | 0.0670 | -2.95 |
| hsa-miR-502-3p | 0.92 | 0.9176 | -1.08 |
| hsa-miR-502 | 1.93 | 0.2472 | 1.93 |
| hsa-miR-503 | 0.67 | 0.6519 | -1.49 |
| hsa-miR-504 | 1.63 | 0.7076 | 1.63 |
| hsa-miR-505 | 1.93 | 0.2472 | 1.93 |
| hsa-miR-507 | 1.93 | 0.2472 | 1.93 |
| hsa-miR-508 | 1.17 | 0.8256 | 1.17 |
| hsa-miR-508-5p | 1.45 | 0.6979 | 1.45 |
| hsa-miR-509-5p | 1.93 | 0.2472 | 1.93 |
| hsa-miR-510 | 1.93 | 0.2472 | 1.93 |
| hsa-miR-512-3p | 1.93 | 0.2472 | 1.93 |
| hsa-miR-512-5p | 1.93 | 0.2472 | 1.93 |
| hsa-miR-513-5p | 1.93 | 0.2472 | 1.93 |
| hsa-miR-515-3p | 1.93 | 0.2472 | 1.93 |
| hsa-miR-515-5p | 1.93 | 0.2472 | 1.93 |
| hsa-miR-516a-5p | 1.93 | 0.2472 | 1.93 |
| hsa-miR-516b | 1.93 | 0.2472 | 1.93 |
| hsa-miR-517a | 1.93 | 0.2472 | 1.93 |
| hsa-miR-517c | 1.57 | 0.3814 | 1.57 |
| hsa-miR-518a-3p | 1.93 | 0.2472 | 1.93 |
| hsa-miR-518a-5p | 1.93 | 0.2472 | 1.93 |
| hsa-miR-518b | 1.15 | 0.8079 | 1.15 |
| hsa-miR-518c | 1.93 | 0.2472 | 1.93 |
| hsa-miR-518d | 2.27 | 0.3926 | 2.27 |
| hsa-miR-518d-5p | 1.93 | 0.2472 | 1.93 |
| hsa-miR-518e | 1.93 | 0.2472 | 1.93 |
| hsa-miR-518f | 0.85 | 0.8395 | -1.17 |
| hsa-miR-519a | 1.22 | 0.7357 | 1.22 |
| hsa-miR-519d | 1.93 | 0.2472 | 1.93 |
| hsa-miR-519e | 1.93 | 0.2472 | 1.93 |
| hsa-miR-520a | 1.93 | 0.2472 | 1.93 |
| hsa-miR-520a# | 1.93 | 0.2472 | 1.93 |
| hsa-miR-520d-5p | **3.08** | 0.3940 | **3.08** |
| hsa-miR-520g | 1.93 | 0.2472 | 1.93 |
| hsa-miR-521 | 1.93 | 0.2472 | 1.93 |
| hsa-miR-522 | 1.92 | 0.2550 | 1.92 |
| hsa-miR-523 | 0.95 | 0.9488 | -1.05 |
| hsa-miR-524-5p | 1.93 | 0.2472 | 1.93 |
| hsa-miR-525-3p | 1.93 | 0.2472 | 1.93 |
| hsa-miR-525 | 1.93 | 0.2472 | 1.93 |
| hsa-miR-526b | 1.93 | 0.2472 | 1.93 |
| hsa-miR-532-3p | 0.71 | 0.5185 | -1.41 |
| hsa-miR-532 | **0.25** | 0.6155 | **-3.98** |
| hsa-miR-539 | **0.11** | 0.1481 | **-9.02** |
| hsa-miR-541 | 1.93 | 0.2472 | 1.93 |
| hsa-miR-542-3p | 0.46 | 0.3228 | -2.18 |
| hsa-miR-542-5p | **0.11** | **0.0431** | **-9.35** |
| hsa-miR-544 | 1.93 | 0.2472 | 1.93 |
| hsa-miR-545 | 0.77 | 0.8100 | -1.30 |
| hsa-miR-548a | 1.96 | 0.3138 | 1.96 |
| hsa-miR-548a-5p | 1.93 | 0.2472 | 1.93 |
| hsa-miR-548b | 1.93 | 0.2472 | 1.93 |
| hsa-miR-548b-5p | 1.93 | 0.2472 | 1.93 |
| hsa-miR-548c | 1.94 | 0.2557 | 1.94 |
| hsa-miR-548c-5p | 1.93 | 0.2472 | 1.93 |
| hsa-miR-548d | 1.93 | 0.2472 | 1.93 |
| hsa-miR-548d-5p | 1.93 | 0.2472 | 1.93 |
| hsa-miR-551b | 1.93 | 0.2472 | 1.93 |
| hsa-miR-556-3p | 1.93 | 0.2472 | 1.93 |
| hsa-miR-556-5p | 1.93 | 0.2472 | 1.93 |
| hsa-miR-561 | 0.77 | 0.7839 | -1.29 |
| hsa-miR-570 | 1.61 | 0.4002 | 1.61 |
| hsa-miR-574-3p | 1.43 | 0.5288 | 1.43 |
| hsa-miR-576-3p | **0.21** | 0.2985 | **-4.77** |
| hsa-miR-576-5p | 1.93 | 0.2472 | 1.93 |
| hsa-miR-579 | 0.43 | 0.1634 | -2.30 |
| hsa-miR-582-3p | 0.90 | 0.9114 | -1.11 |
| hsa-miR-582-5p | 0.72 | 0.7024 | -1.39 |
| hsa-miR-589 | 1.93 | 0.2472 | 1.93 |
| hsa-miR-590-5p | 0.85 | 0.7661 | -1.18 |
| hsa-miR-597 | 0.74 | 0.6218 | -1.34 |
| hsa-miR-598 | 2.07 | 0.2181 | 2.07 |
| mmu-miR-615 | 1.93 | 0.2472 | 1.93 |
| hsa-miR-615-5p | 1.93 | 0.2472 | 1.93 |
| hsa-miR-616 | **4.87** | **0.0466** | **4.87** |
| hsa-miR-618 | 0.63 | 0.7638 | -1.59 |
| hsa-miR-624 | 1.93 | 0.2472 | 1.93 |
| hsa-miR-625 | 0.53 | 0.2290 | -1.89 |
| hsa-miR-627 | 1.92 | 0.7030 | 1.92 |
| hsa-miR-628-5p | 1.44 | 0.6471 | 1.44 |
| hsa-miR-629 | 1.93 | 0.2472 | 1.93 |
| hsa-miR-636 | 1.61 | 0.6229 | 1.61 |
| hsa-miR-642 | 1.37 | 0.6140 | 1.37 |
| hsa-miR-651 | 1.93 | 0.2472 | 1.93 |
| hsa-miR-652 | **0.21** | 0.1015 | **-4.88** |
| hsa-miR-653 | 1.93 | 0.2472 | 1.93 |
| hsa-miR-654-3p | 1.91 | 0.2503 | 1.91 |
| hsa-miR-654 | 1.31 | 0.6733 | 1.31 |
| hsa-miR-655 | 0.42 | 0.3888 | -2.41 |
| hsa-miR-660 | **0.20** | 0.4932 | **-5.11** |
| hsa-miR-671-3p | 0.84 | 0.8913 | -1.19 |
| hsa-miR-672 | **3.10** | 0.2363 | **3.10** |
| hsa-miR-674 | 1.93 | 0.2472 | 1.93 |
| hsa-miR-708 | 1.93 | 0.2472 | 1.93 |
| hsa-miR-744 | 0.38 | 0.3618 | -2.62 |
| hsa-miR-758 | 1.17 | 0.8111 | 1.17 |
| hsa-miR-871 | 1.93 | 0.2472 | 1.93 |
| hsa-miR-872 | 1.93 | 0.2472 | 1.93 |
| hsa-miR-873 | 1.93 | 0.2472 | 1.93 |
| hsa-miR-874 | 1.93 | 0.2472 | 1.93 |
| hsa-miR-875-3p | 1.93 | 0.2472 | 1.93 |
| hsa-miR-876-3p | 1.93 | 0.2472 | 1.93 |
| hsa-miR-876-5p | 1.93 | 0.2472 | 1.93 |
| hsa-miR-885-3p | 0.83 | 0.8538 | -1.20 |
| hsa-miR-885-5p | 1.56 | 0.4463 | 1.56 |
| hsa-miR-886-3p | 1.39 | 0.1885 | 1.39 |
| hsa-miR-886-5p | 1.61 | 0.2592 | 1.61 |
| hsa-miR-887 | 1.93 | 0.2472 | 1.93 |
| hsa-miR-888 | 0.42 | 0.6978 | -2.41 |
| hsa-miR-889 | 0.63 | 0.6228 | -1.60 |
| hsa-miR-890 | 1.93 | 0.2472 | 1.93 |
| hsa-miR-891a | 1.93 | 0.2472 | 1.93 |
| hsa-miR-891b | 1.93 | 0.2472 | 1.93 |
| hsa-miR-892a | 1.93 | 0.2472 | 1.93 |
| hsa-miR-147 | 1.93 | 0.2472 | 1.93 |
| hsa-miR-208 | 1.93 | 0.2472 | 1.93 |
| hsa-miR-211 | 1.93 | 0.2472 | 1.93 |
| hsa-miR-212 | **0.29** | 0.5572 | **-3.41** |
| hsa-miR-219-1-3p | **5.09** | 0.1176 | **5.09** |
| hsa-miR-219-2-3p | 2.42 | **0.0419** | 2.42 |
| hsa-miR-220 | 1.93 | 0.2472 | 1.93 |
| hsa-miR-220b | 1.93 | 0.2472 | 1.93 |
| hsa-miR-220c | 1.93 | 0.2472 | 1.93 |
| hsa-miR-298 | 1.93 | 0.2472 | 1.93 |
| hsa-miR-325 | 1.21 | 0.7472 | 1.21 |
| hsa-miR-346 | 1.93 | 0.2472 | 1.93 |
| hsa-miR-376c | **0.13** | 0.3070 | **-7.73** |
| hsa-miR-384 | 1.93 | 0.2472 | 1.93 |
| hsa-miR-412 | 1.87 | 0.2666 | 1.87 |
| hsa-miR-448 | 1.93 | 0.2472 | 1.93 |
| hsa-miR-492 | 1.93 | 0.2472 | 1.93 |
| hsa-miR-506 | 1.93 | 0.2472 | 1.93 |
| hsa-miR-509-3-5p | 1.93 | 0.2472 | 1.93 |
| hsa-miR-511 | 1.93 | 0.2472 | 1.93 |
| hsa-miR-517b | 2.74 | 0.2337 | 2.74 |
| hsa-miR-519c | 1.93 | 0.2472 | 1.93 |
| hsa-miR-520b | 1.93 | 0.2472 | 1.93 |
| hsa-miR-520e | 1.93 | 0.2472 | 1.93 |
| hsa-miR-520f | 1.93 | 0.2472 | 1.93 |
| dme-miR-7 | **3.42** | 0.1805 | **3.42** |
| hsa-miR-548I | **3.42** | 0.1805 | **3.42** |
| hsa-miR-30a-3p | 1.82 | 0.5565 | 1.82 |
| hsa-miR-30a-5p | 0.51 | 0.4922 | -1.96 |
| hsa-miR-30d | 0.63 | 0.4958 | -1.58 |
| hsa-miR-30e-3p | **0.30** | 0.4973 | **-3.29** |
| hsa-miR-34b | **3.42** | 0.1805 | **3.42** |
| hsa-miR-126# | 1.44 | 0.7502 | 1.44 |
| hsa-miR-154# | **3.42** | 0.1805 | **3.42** |
| hsa-miR-182# | **3.42** | 0.1805 | **3.42** |
| U6 snRNA -1 | 1.03 | 0.7051 | 1.03 |
| U6 snRNA -2 | 1.10 | 0.2903 | 1.10 |
| hsa-miR-206 | 1.71 | 0.5860 | 1.71 |
| hsa-miR-213 | 0.37 | 0.2648 | -2.72 |
| hsa-miR-302c# | **3.42** | 0.1805 | **3.42** |
| hsa-miR-302d | 2.71 | 0.2221 | 2.71 |
| hsa-miR-378 | **3.42** | 0.1805 | **3.42** |
| hsa-miR-380-5p | 2.51 | 0.3073 | 2.51 |
| hsa-miR-1257 | **3.42** | 0.1805 | **3.42** |
| hsa-miR-200a# | **3.47** | 0.1719 | **3.47** |
| hsa-miR-432 | 2.16 | 0.5489 | 2.16 |
| hsa-miR-432# | **3.42** | 0.1805 | **3.42** |
| hsa-miR-497 | 0.86 | 0.8916 | -1.17 |
| hsa-miR-500 | 2.91 | 0.2356 | 2.91 |
| hsa-miR-1238 | **3.42** | 0.1805 | **3.42** |
| hsa-miR-488 | **3.42** | 0.1805 | **3.42** |
| hsa-miR-517# | **3.42** | 0.1805 | **3.42** |
| hsa-miR-516-3p | 2.72 | 0.2466 | 2.72 |
| hsa-miR-518c# | **3.42** | 0.1805 | **3.42** |
| hsa-miR-519e# | **4.31** | 0.1818 | **4.31** |
| hsa-miR-520h | **3.42** | 0.1805 | **3.42** |
| hsa-miR-524 | **3.42** | 0.1805 | **3.42** |
| mmu-let-7d# | **3.42** | 0.1805 | **3.42** |
| hsa-miR-363# | **3.42** | 0.1805 | **3.42** |
| U6 snRNA-3 | 0.88 | 0.0859 | -1.13 |
| U6 snRNA -4 | 1.00 | 0.9912 | 1.00 |
| rno-miR-7# | 1.30 | 0.8000 | 1.30 |
| hsa-miR-656 | 1.59 | 0.7395 | 1.59 |
| hsa-miR-549 | **8.86** | **0.0187** | **8.86** |
| hsa-miR-657 | **3.42** | 0.1805 | **3.42** |
| hsa-miR-658 | **3.42** | 0.1805 | **3.42** |
| hsa-miR-659 | **3.42** | 0.1805 | **3.42** |
| hsa-miR-551a | 1.92 | 0.5091 | 1.92 |
| hsa-miR-552 | **3.42** | 0.1805 | **3.42** |
| hsa-miR-553 | **3.42** | 0.1805 | **3.42** |
| hsa-miR-554 | **3.42** | 0.1805 | **3.42** |
| hsa-miR-555 | **3.42** | 0.1805 | **3.42** |
| hsa-miR-557 | **3.42** | 0.1805 | **3.42** |
| hsa-miR-558 | **3.42** | 0.1805 | **3.42** |
| hsa-miR-559 | **3.42** | 0.1805 | **3.42** |
| hsa-miR-562 | **3.42** | 0.1805 | **3.42** |
| hsa-miR-563 | 2.16 | 0.3309 | 2.16 |
| hsa-miR-564 | 0.60 | 0.5792 | -1.67 |
| hsa-miR-566 | 2.54 | 0.3009 | 2.54 |
| hsa-miR-567 | **3.42** | 0.1805 | **3.42** |
| hsa-miR-569 | **3.42** | 0.1805 | **3.42** |
| hsa-miR-586 | 1.78 | 0.7435 | 1.78 |
| hsa-miR-587 | **3.42** | 0.1805 | **3.42** |
| RNU44 | 2.33 | 0.3418 | 2.33 |
| hsa-miR-588 | **3.42** | 0.1805 | **3.42** |
| hsa-miR-589 | **4.86** | 0.1778 | **4.86** |
| hsa-miR-550 | 1.83 | 0.7656 | 1.83 |
| hsa-miR-591 | **3.42** | 0.1805 | **3.42** |
| hsa-miR-592 | **3.42** | 0.1805 | **3.42** |
| hsa-miR-593 | **3.42** | 0.1805 | **3.42** |
| hsa-miR-596 | 2.89 | 0.2864 | 2.89 |
| hsa-miR-622 | **3.42** | 0.1805 | **3.42** |
| hsa-miR-599 | **3.42** | 0.1805 | **3.42** |
| hsa-miR-623 | **3.42** | 0.1805 | **3.42** |
| hsa-miR-600 | **3.42** | 0.1805 | **3.42** |
| hsa-miR-624 | **3.06** | 0.2128 | **3.06** |
| hsa-miR-601 | 2.17 | 0.6180 | 2.17 |
| hsa-miR-626 | **3.42** | 0.1805 | **3.42** |
| hsa-miR-629 | **9.76** | 0.0871 | **9.76** |
| hsa-miR-630 | 2.15 | 0.3329 | 2.15 |
| hsa-miR-631 | **3.42** | 0.1805 | **3.42** |
| hsa-miR-603 | 2.15 | 0.2788 | 2.15 |
| hsa-miR-604 | 1.34 | 0.6685 | 1.34 |
| hsa-miR-605 | **3.42** | 0.1805 | **3.42** |
| hsa-miR-606 | **3.42** | 0.1805 | **3.42** |
| hsa-miR-607 | **3.42** | 0.1805 | **3.42** |
| hsa-miR-608 | **3.42** | 0.1805 | **3.42** |
| hsa-miR-609 | **3.42** | 0.1805 | **3.42** |
| hsa-miR-633 | **3.42** | 0.1805 | **3.42** |
| hsa-miR-634 | **3.42** | 0.1805 | **3.42** |
| hsa-miR-635 | **3.42** | 0.1805 | **3.42** |
| hsa-miR-637 | **3.42** | 0.1805 | **3.42** |
| hsa-miR-638 | **4.12** | 0.1621 | **4.12** |
| hsa-miR-639 | **3.42** | 0.1805 | **3.42** |
| hsa-miR-640 | **3.42** | 0.1805 | **3.42** |
| hsa-miR-641 | **3.42** | 0.1805 | **3.42** |
| hsa-miR-613 | **3.42** | 0.1805 | **3.42** |
| hsa-miR-614 | **3.42** | 0.1805 | **3.42** |
| hsa-miR-616 | 2.39 | 0.3520 | 2.39 |
| hsa-miR-617 | 2.49 | 0.3137 | 2.49 |
| hsa-miR-643 | **3.42** | 0.1805 | **3.42** |
| hsa-miR-644 | **3.42** | 0.1805 | **3.42** |
| hsa-miR-645 | **3.42** | 0.1805 | **3.42** |
| hsa-miR-621 | **3.42** | 0.1805 | **3.42** |
| hsa-miR-646 | **3.42** | 0.1805 | **3.42** |
| hsa-miR-647 | **3.42** | 0.1805 | **3.42** |
| hsa-miR-648 | **3.42** | 0.1805 | **3.42** |
| hsa-miR-649 | 0.66 | 0.6563 | -1.52 |
| hsa-miR-650 | 2.58 | 0.4746 | 2.58 |
| hsa-miR-661 | 0.54 | 0.6483 | -1.84 |
| hsa-miR-662 | **3.42** | 0.1805 | **3.42** |
| RNU48 | 2.73 | 0.1577 | 2.73 |
| hsa-miR-571 | **3.42** | 0.1805 | **3.42** |
| hsa-miR-572 | 2.63 | 0.2809 | 2.63 |
| hsa-miR-573 | **3.42** | 0.1805 | **3.42** |
| hsa-miR-575 | **3.42** | 0.1805 | **3.42** |
| hsa-miR-578 | **3.42** | 0.1805 | **3.42** |
| hsa-miR-580 | **3.42** | 0.1805 | **3.42** |
| hsa-miR-581 | **3.42** | 0.1805 | **3.42** |
| hsa-miR-583 | **3.42** | 0.1805 | **3.42** |
| hsa-miR-584 | **3.42** | 0.1805 | **3.42** |
| hsa-miR-585 | **3.42** | 0.1805 | **3.42** |
| rno-miR-29c# | 1.37 | 0.7461 | 1.37 |
| hsa-miR-766 | 2.39 | 0.2584 | 2.39 |
| hsa-miR-595 | **3.42** | 0.1805 | **3.42** |
| hsa-miR-668 | **3.42** | 0.1805 | **3.42** |
| hsa-miR-767-5p | **3.42** | 0.1805 | **3.42** |
| hsa-miR-767-3p | **21.90** | 0.0808 | **21.90** |
| hsa-miR-454# | 2.58 | 0.4003 | 2.58 |
| hsa-miR-769-5p | **0.31** | 0.2912 | **-3.28** |
| hsa-miR-770-5p | 0.73 | 0.7367 | -1.38 |
| hsa-miR-769-3p | **3.42** | 0.1805 | **3.42** |
| hsa-miR-802 | **4.35** | 0.0661 | **4.35** |
| hsa-miR-675 | **3.42** | 0.1805 | **3.42** |
| hsa-miR-505# | 0.91 | 0.8988 | -1.10 |
| hsa-miR-218-1# | **3.42** | 0.1805 | **3.42** |
| hsa-miR-221# | **3.19** | 0.1988 | **3.19** |
| hsa-miR-222# | 0.82 | 0.8447 | -1.21 |
| hsa-miR-223# | 0.98 | 0.9830 | -1.02 |
| hsa-miR-136# | **0.25** | 0.2990 | **-4.07** |
| hsa-miR-34b | **3.10** | 0.2382 | **3.10** |
| hsa-miR-185# | **3.42** | 0.1805 | **3.42** |
| hsa-miR-186# | **3.42** | 0.1805 | **3.42** |
| hsa-miR-195# | **3.42** | 0.1805 | **3.42** |
| hsa-miR-30c-1# | **3.42** | 0.1805 | **3.42** |
| hsa-miR-30c-2# | **3.42** | 0.1805 | **3.42** |
| hsa-miR-32# | **3.42** | 0.1805 | **3.42** |
| hsa-miR-31# | 2.94 | 0.2746 | 2.94 |
| hsa-miR-130b# | 2.52 | 0.3911 | 2.52 |
| hsa-miR-26a-2# | 2.89 | 0.2347 | 2.89 |
| hsa-miR-361-3p | 2.60 | 0.2879 | 2.60 |
| hsa-let-7g# | 2.73 | 0.2201 | 2.73 |
| hsa-miR-302b# | **3.42** | 0.1805 | **3.42** |
| hsa-miR-302d# | **3.42** | 0.1805 | **3.42** |
| hsa-miR-367# | **3.42** | 0.1805 | **3.42** |
| hsa-miR-374a# | **3.42** | 0.1805 | **3.42** |
| hsa-miR-23b# | **3.42** | 0.1805 | **3.42** |
| hsa-miR-376a# | 2.70 | 0.2230 | 2.70 |
| hsa-miR-377# | **3.42** | 0.1805 | **3.42** |
| ath-miR159a | **3.42** | 0.1805 | **3.42** |
| hsa-miR-30b# | **3.42** | 0.1805 | **3.42** |
| hsa-miR-122# | 1.50 | 0.7234 | 1.50 |
| hsa-miR-130a# | 1.75 | 0.5902 | 1.75 |
| hsa-miR-132# | **3.42** | 0.1805 | **3.42** |
| hsa-miR-148a# | **3.42** | 0.1805 | **3.42** |
| hsa-miR-33a | **4.33** | 0.1603 | **4.33** |
| hsa-miR-33a# | 0.73 | 0.7851 | -1.38 |
| hsa-miR-92a-1# | 1.72 | 0.4885 | 1.72 |
| hsa-miR-92a-2# | **3.42** | 0.1805 | **3.42** |
| hsa-miR-93# | **0.21** | 0.6268 | **-4.65** |
| hsa-miR-96# | **3.42** | 0.1805 | **3.42** |
| hsa-miR-99a# | **3.42** | 0.1805 | **3.42** |
| hsa-miR-100# | 1.70 | 0.5304 | 1.70 |
| hsa-miR-101# | 2.58 | 0.2927 | 2.58 |
| hsa-miR-138-2# | **3.42** | 0.1805 | **3.42** |
| hsa-miR-141# | **3.07** | 0.2118 | **3.07** |
| hsa-miR-143# | **3.42** | 0.1805 | **3.42** |
| hsa-miR-144# | **11.06** | 0.1006 | **11.06** |
| hsa-miR-145# | **3.13** | 0.3709 | **3.13** |
| hsa-miR-920 | **3.42** | 0.1805 | **3.42** |
| hsa-miR-921 | **3.42** | 0.1805 | **3.42** |
| hsa-miR-922 | **3.42** | 0.1805 | **3.42** |
| hsa-miR-924 | **3.42** | 0.1805 | **3.42** |
| hsa-miR-337-3p | **3.42** | 0.1805 | **3.42** |
| hsa-miR-125b-2# | **3.42** | 0.1805 | **3.42** |
| hsa-miR-135b# | **3.42** | 0.1805 | **3.42** |
| hsa-miR-148b# | 1.69 | 0.6958 | 1.69 |
| hsa-miR-146a# | **3.42** | 0.1805 | **3.42** |
| hsa-miR-149# | **3.42** | 0.1805 | **3.42** |
| hsa-miR-29b-1# | **3.42** | 0.1805 | **3.42** |
| hsa-miR-29b-2# | **3.42** | 0.1805 | **3.42** |
| hsa-miR-105# | **3.42** | 0.1805 | **3.42** |
| hsa-miR-106a# | **3.42** | 0.1805 | **3.42** |
| hsa-miR-16-2# | 2.33 | 0.4769 | 2.33 |
| hsa-let-7i# | 2.47 | 0.2938 | 2.47 |
| hsa-miR-15b# | 0.38 | 0.6902 | -2.62 |
| hsa-miR-27b# | **3.42** | 0.1805 | **3.42** |
| hsa-miR-933 | **3.42** | 0.1805 | **3.42** |
| hsa-miR-934 | **3.42** | 0.1805 | **3.42** |
| hsa-miR-935 | **3.42** | 0.1805 | **3.42** |
| hsa-miR-936 | **3.42** | 0.1805 | **3.42** |
| hsa-miR-937 | **3.42** | 0.1805 | **3.42** |
| hsa-miR-938 | **4.42** | 0.1173 | **4.42** |
| hsa-miR-939 | 2.10 | 0.7227 | 2.10 |
| hsa-miR-941 | **4.05** | 0.2686 | **4.05** |
| hsa-miR-335# | 1.10 | 0.9435 | 1.10 |
| hsa-miR-942 | **4.87** | 0.1690 | **4.87** |
| hsa-miR-943 | **3.42** | 0.1805 | **3.42** |
| hsa-miR-944 | **3.42** | 0.1805 | **3.42** |
| hsa-miR-99b# | **3.93** | 0.1092 | **3.93** |
| hsa-miR-124# | **3.42** | 0.1805 | **3.42** |
| hsa-miR-541# | **3.42** | 0.1805 | **3.42** |
| hsa-miR-875-5p | **6.68** | 0.4431 | **6.68** |
| hsa-miR-888# | **3.42** | 0.1805 | **3.42** |
| hsa-miR-892b | **3.42** | 0.1805 | **3.42** |
| hsa-miR-9# | **3.42** | 0.1805 | **3.42** |
| hsa-miR-411# | **3.42** | 0.1805 | **3.42** |
| hsa-miR-378 | 0.33 | 0.6334 | **-3.00** |
| hsa-miR-151-3p | **36.94** | 0.1761 | **36.94** |
| hsa-miR-340# | 0.88 | 0.9206 | -1.14 |
| hsa-miR-190b | 1.19 | 0.8111 | 1.19 |
| hsa-miR-545# | **3.42** | 0.1805 | **3.42** |
| hsa-miR-183# | **5.45** | 0.0826 | **5.45** |
| hsa-miR-192# | 2.73 | 0.3297 | 2.73 |
| hsa-miR-200b# | **3.42** | 0.1805 | **3.42** |
| hsa-miR-200c# | **3.42** | 0.1805 | **3.42** |
| hsa-miR-155# | **3.42** | 0.1805 | **3.42** |
| hsa-miR-10a# | **3.42** | 0.1805 | **3.42** |
| hsa-miR-214# | **3.42** | 0.1805 | **3.42** |
| hsa-miR-218-2# | **3.42** | 0.1805 | **3.42** |
| hsa-miR-129# | **3.42** | 0.1805 | **3.42** |
| hsa-miR-22# | 2.62 | 0.2840 | 2.62 |
| hsa-miR-425# | **3.60** | 0.3591 | **3.60** |
| hsa-miR-30d# | 1.81 | 0.6717 | 1.81 |
| hsa-let-7a# | **3.42** | 0.1805 | **3.42** |
| hsa-miR-424# | **3.78** | 0.3648 | **3.78** |
| hsa-miR-18b# | **3.42** | 0.1805 | **3.42** |
| hsa-miR-20b# | **3.42** | 0.1805 | **3.42** |
| hsa-miR-431# | **3.42** | 0.1805 | **3.42** |
| hsa-miR-7-2# | **3.42** | 0.1805 | **3.42** |
| hsa-miR-10b# | 1.36 | 0.7484 | 1.36 |
| hsa-miR-34a# | 1.50 | 0.7414 | 1.50 |
| hsa-miR-181a-2# | 0.72 | 0.5700 | -1.38 |
| hsa-miR-744# | 1.30 | 0.8310 | 1.30 |
| hsa-miR-452# | **3.42** | 0.1805 | **3.42** |
| hsa-miR-409-3p | **0.11** | 0.2244 | **-8.73** |
| hsa-miR-181c# | 1.93 | 0.5048 | 1.93 |
| hsa-miR-196a# | **3.42** | 0.1805 | **3.42** |
| hsa-miR-483-3p | **3.42** | 0.1805 | **3.42** |
| hsa-miR-708# | **3.42** | 0.1805 | **3.42** |
| hsa-miR-92b# | **3.42** | 0.1805 | **3.42** |
| hsa-miR-551b# | **3.42** | 0.1805 | **3.42** |
| hsa-miR-202# | **3.42** | 0.1805 | **3.42** |
| hsa-miR-193b# | **3.42** | 0.1805 | **3.42** |
| hsa-miR-497# | **3.42** | 0.1805 | **3.42** |
| hsa-miR-518e# | **3.42** | 0.1805 | **3.42** |
| hsa-miR-543 | **3.42** | 0.1805 | **3.42** |
| hsa-miR-125b-1# | **3.42** | 0.1805 | **3.42** |
| hsa-miR-194# | **3.42** | 0.1805 | **3.42** |
| hsa-miR-106b# | 1.17 | 0.9181 | 1.17 |
| hsa-miR-302a# | **3.42** | 0.1805 | **3.42** |
| hsa-miR-519b-3p | **3.42** | 0.1805 | **3.42** |
| hsa-miR-518f# | **3.42** | 0.1805 | **3.42** |
| hsa-miR-374b# | **3.42** | 0.1805 | **3.42** |
| hsa-miR-520c-3p | 1.73 | 0.5748 | 1.73 |
| hsa-let-7b# | **3.42** | 0.1805 | **3.42** |
| hsa-let-7c# | **3.42** | 0.1805 | **3.42** |
| hsa-let-7e# | **3.42** | 0.1805 | **3.42** |
| hsa-miR-550 | 2.78 | 0.5539 | 2.78 |
| hsa-miR-593 | **3.42** | 0.1805 | **3.42** |
| hsa-let-7f-1# | **3.42** | 0.1805 | **3.42** |
| hsa-let-7f-2# | **5.45** | 0.1928 | **5.45** |
| hsa-miR-15a# | 0.80 | 0.8003 | -1.25 |
| hsa-miR-16-1# | 0.56 | 0.5142 | -1.79 |
| hsa-miR-17# | 1.16 | 0.8989 | 1.16 |
| hsa-miR-18a# | 1.73 | 0.5357 | 1.73 |
| hsa-miR-19a# | **3.42** | 0.1805 | **3.42** |
| hsa-miR-19b-1# | 0.73 | 0.8817 | -1.36 |
| hsa-miR-625# | 2.90 | 0.3874 | 2.90 |
| hsa-miR-628-3p | **3.37** | 0.2979 | **3.37** |
| hsa-miR-20a# | 1.64 | 0.5240 | 1.64 |
| hsa-miR-21# | 1.71 | 0.5280 | 1.71 |
| hsa-miR-23a# | **3.42** | 0.1805 | **3.42** |
| hsa-miR-24-1# | **3.42** | 0.1805 | **3.42** |
| hsa-miR-24-2# | 0.53 | 0.5841 | -1.88 |
| hsa-miR-25# | **3.42** | 0.1805 | **3.42** |
| hsa-miR-26a-1# | 2.51 | 0.6311 | 2.51 |
| hsa-miR-26b# | 2.40 | 0.2556 | 2.40 |
| hsa-miR-27a# | 1.11 | 0.9108 | 1.11 |
| hsa-miR-29a# | 2.29 | 0.3774 | 2.29 |
| hsa-miR-151-5P | **4.39** | 0.0631 | **4.39** |
| hsa-miR-765 | **3.42** | 0.1805 | **3.42** |
| hsa-miR-338-5P | 1.38 | 0.6257 | 1.38 |
| hsa-miR-620 | **3.42** | 0.1805 | **3.42** |
| hsa-miR-577 | 1.91 | 0.5361 | 1.91 |
| hsa-miR-144 | **12.30** | **0.0468** | **12.30** |
| hsa-miR-590-3P | **4.32** | 0.0525 | **4.32** |
| hsa-miR-191# | 1.42 | 0.6238 | 1.42 |
| hsa-miR-665 | **3.42** | 0.1805 | **3.42** |
| hsa-miR-520D-3P | 1.37 | 0.7461 | 1.37 |
| hsa-miR-1224-3P | **3.42** | 0.1805 | **3.42** |
| hsa-miR-1305 | **5.44** | **0.0239** | **5.44** |
| hsa-miR-513C | **3.42** | 0.1805 | **3.42** |
| hsa-miR-513B | **3.42** | 0.1805 | **3.42** |
| hsa-miR-1226# | **3.24** | 0.2792 | **3.24** |
| hsa-miR-1236 | **3.42** | 0.1805 | **3.42** |
| hsa-miR-1228# | **3.42** | 0.1805 | **3.42** |
| hsa-miR-1225-3P | **3.42** | 0.1805 | **3.42** |
| hsa-miR-1233 | **3.94** | 0.4573 | **3.94** |
| hsa-miR-1227 | 1.88 | 0.5331 | 1.88 |
| hsa-miR-1286 | **3.42** | 0.1805 | **3.42** |
| hsa-miR-548M | **3.42** | 0.1805 | **3.42** |
| hsa-miR-1179 | **3.76** | 0.1283 | **3.76** |
| hsa-miR-1178 | **3.42** | 0.1805 | **3.42** |
| hsa-miR-1205 | **3.42** | 0.1805 | **3.42** |
| hsa-miR-1271 | 1.66 | 0.4146 | 1.66 |
| hsa-miR-1201 | 1.39 | 0.7845 | 1.39 |
| hsa-miR-548J | **3.42** | 0.1805 | **3.42** |
| hsa-miR-1263 | **3.42** | 0.1805 | **3.42** |
| hsa-miR-1294 | **3.42** | 0.1805 | **3.42** |
| hsa-miR-1269 | **3.42** | 0.1805 | **3.42** |
| hsa-miR-1265 | **3.42** | 0.1805 | **3.42** |
| hsa-miR-1244 | 1.03 | 0.9626 | 1.03 |
| hsa-miR-1303 | **3.42** | 0.1805 | **3.42** |
| hsa-miR-1259 | **3.42** | 0.1805 | **3.42** |
| hsa-miR-548P | **3.42** | 0.1805 | **3.42** |
| hsa-miR-1264 | **3.42** | 0.1805 | **3.42** |
| hsa-miR-1255B | 1.23 | 0.9052 | 1.23 |
| hsa-miR-1282 | **3.53** | 0.1759 | **3.53** |
| hsa-miR-1255A | **3.42** | 0.1805 | **3.42** |
| hsa-miR-1270 | **3.42** | 0.1805 | **3.42** |
| hsa-miR-1197 | **3.42** | 0.1805 | **3.42** |
| hsa-miR-1324 | **3.42** | 0.1805 | **3.42** |
| hsa-miR-548H | **3.42** | 0.1805 | **3.42** |
| hsa-miR-1254 | 0.85 | 0.7802 | -1.18 |
| hsa-miR-548K | **3.42** | 0.1805 | **3.42** |
| hsa-miR-1251 | **3.42** | 0.1805 | **3.42** |
| hsa-miR-1285 | 1.65 | 0.7020 | 1.65 |
| hsa-miR-1245 | **3.42** | 0.1805 | **3.42** |
| hsa-miR-1292 | **3.42** | 0.1805 | **3.42** |
| hsa-miR-1301 | **3.42** | 0.1805 | **3.42** |
| hsa-miR-1200 | **3.42** | 0.1805 | **3.42** |
| hsa-miR-1182 | **3.42** | 0.1805 | **3.42** |
| hsa-miR-1288 | **3.42** | 0.1805 | **3.42** |
| hsa-miR-1291 | 0.39 | 0.3747 | -2.57 |
| hsa-miR-1275 | 0.94 | 0.9374 | -1.07 |
| hsa-miR-1183 | 1.35 | 0.7980 | 1.35 |
| hsa-miR-1184 | **3.42** | 0.1805 | **3.42** |
| hsa-miR-1276 | 2.15 | 0.3330 | 2.15 |
| hsa-miR-320B | 0.86 | 0.8819 | -1.16 |
| hsa-miR-1272 | **3.42** | 0.1805 | **3.42** |
| hsa-miR-1180 | **3.42** | 0.1805 | **3.42** |
| hsa-miR-1256 | **3.42** | 0.1805 | **3.42** |
| hsa-miR-1278 | **3.42** | 0.1805 | **3.42** |
| hsa-miR-1262 | 1.36 | 0.8901 | 1.36 |
| hsa-miR-1243 | 2.68 | 0.4455 | 2.68 |
| hsa-miR-663B | **3.42** | 0.1805 | **3.42** |
| hsa-miR-1252 | **3.42** | 0.1805 | **3.42** |
| hsa-miR-1298 | **3.42** | 0.1805 | **3.42** |
| hsa-miR-1290 | **21.62** | 0.2638 | **21.62** |
| hsa-miR-1249 | **4.42** | 0.1827 | **4.42** |
| hsa-miR-1248 | **3.42** | 0.1805 | **3.42** |
| hsa-miR-1289 | **3.42** | 0.1805 | **3.42** |
| hsa-miR-1204 | **3.42** | 0.1805 | **3.42** |
| hsa-miR-1826 | **3.42** | 0.1805 | **3.42** |
| hsa-miR-1304 | **3.42** | 0.1805 | **3.42** |
| hsa-miR-1203 | **3.42** | 0.1805 | **3.42** |
| hsa-miR-1206 | **3.42** | 0.1805 | **3.42** |
| hsa-miR-548G | **3.42** | 0.1805 | **3.42** |
| hsa-miR-1208 | **3.42** | 0.1805 | **3.42** |
| hsa-miR-548E | **3.42** | 0.1805 | **3.42** |
| hsa-miR-1274A | 1.74 | 0.5078 | 1.74 |
| hsa-miR-1274B | 1.07 | 0.9204 | 1.07 |
| hsa-miR-1267 | 0.85 | 0.9013 | -1.18 |
| hsa-miR-1250 | **3.42** | 0.1805 | **3.42** |
| hsa-miR-548N | **3.42** | 0.1805 | **3.42** |
| hsa-miR-1283 | **3.42** | 0.1805 | **3.42** |
| hsa-miR-1247 | **3.42** | 0.1805 | **3.42** |
| hsa-miR-1253 | **3.42** | 0.1805 | **3.42** |
| hsa-miR-720 | 1.57 | 0.6808 | 1.57 |
| hsa-miR-1260 | 2.40 | 0.4953 | 2.40 |
| hsa-miR-664 | **0.26** | 0.6729 | **-3.85** |
| hsa-miR-1302 | **3.42** | 0.1805 | **3.42** |
| hsa-miR-1300 | 1.35 | 0.7575 | 1.35 |
| hsa-miR-1284 | **3.42** | 0.1805 | **3.42** |
| hsa-miR-548L | 2.87 | 0.2381 | 2.87 |
| hsa-miR-1293 | **3.42** | 0.1805 | **3.42** |
| hsa-miR-1825 | **3.42** | 0.1805 | **3.42** |
| hsa-miR-1296 | **3.42** | 0.1805 | **3.42** |
