# Supplementary figures and images for: miR-139-5p Regulates the Proliferation of Acute Promyelocytic Leukemia Cells by Targeting MNT
Source: J Oncol. 2021 Apr 16;2021:5522051. doi: 10.1155/2021/5522051 (PMC8064781; doi:10.1155/2021/5522051)

**Supplemental Table 1. Clinical and laboratory information of patients.**


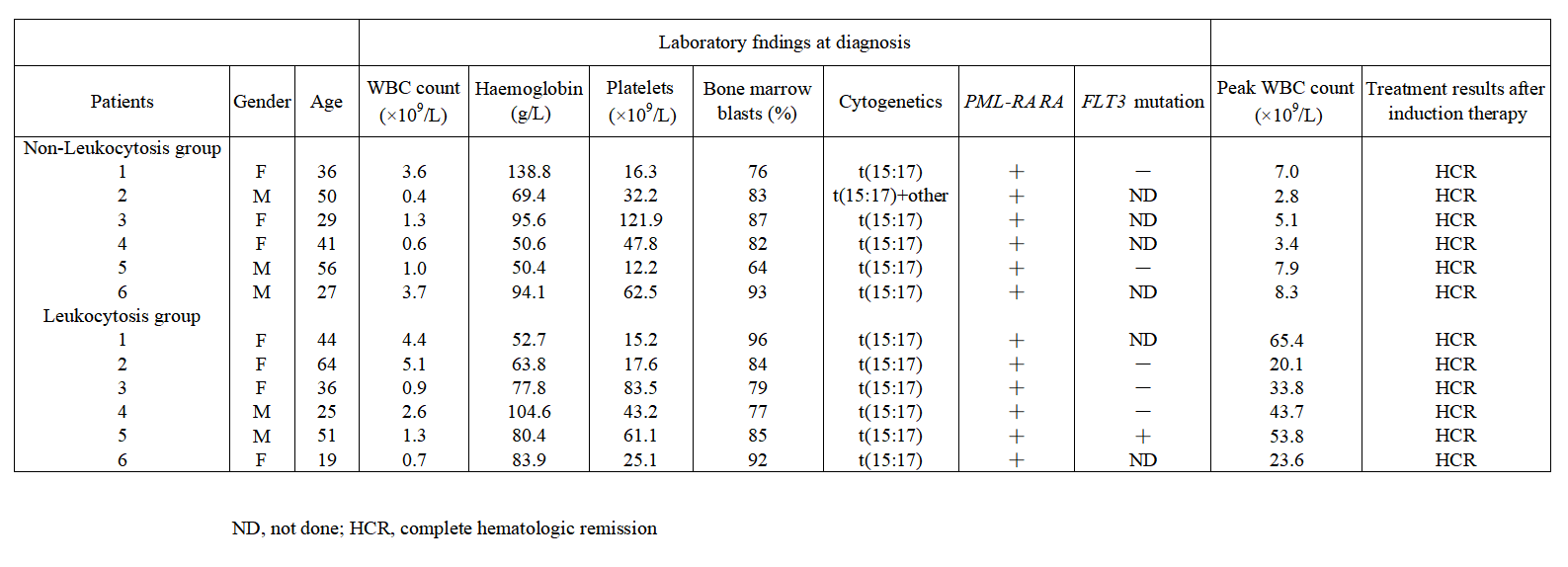

Supplement: Supplementary Materials — Supplemental Table 1: clinical and laboratory information of patients. Supplemental Table 2: data of the quantitative PCR-based array. [file 5522051.f1.zip › 5522051.f1/Supplemental Table 1.docx]
